# Bisphenol A and pubertal height growth in school-aged children

**DOI:** 10.1038/s41370-018-0063-8

**Published:** 2018-09-05

**Authors:** Ziliang Wang, Hong Liang, Xiaowen Tu, Wei Yuan, Zhijun Zhou, Longmei Jin, Maohua Miao, De-Kun Li

**Affiliations:** 1 Key Lab. of Reproduction Regulation of NPFPC, SIPPR, IRD, Fudan University, Shanghai, 200237 China; 2 School of Public Health, Fudan University, Shanghai, 200032 China; 3 School of Public Health, Key Laboratory for Public Health Safety, Fudan University, Shanghai, 200032 China; 4 Minhang Maternal and Child Health Hospital, Shanghai, 201102 China; 5 Division of Research, Kaiser Foundation Research Institute, Kaiser Permanente, Oakland, CA 94612 USA

**Keywords:** Bisphenol A, Endocrine disruptor, Pubertal growth, Height growth

## Abstract

**Background:**

Bisphenol A (BPA) is an environmental endocrine disruptor and is found in many consumer products. Studies suggest that BPA may perturb pubertal development, although evidence on BPA-influenced pubertal height growth is scarce.

**Methods:**

A total of 754 children aged 9–18 years from three schools (one elementary, one middle, and one high school) in Shanghai were included in this longitudinal study. Height was measured at enrolment (visit 1) and, subsequently, at 19 months after enrolment (visit 2). Age- and sex-specific *Z* scores for height were calculated (height *Z* score = [participant’s height−sex- and age-specific population height mean]/sex- and age-specific population height standard deviation). Urine samples were collected at enrolment to measure BPA concentrations. We used multiple linear regression models or general estimating equation models (GEE) to estimate associations between urine BPA level and height *Z* score.

**Results:**

The geometric mean of urine BPA concentrations was 1.6 μg/L (95%CI: 1.4, 1.8) or 1.2 μg/g creatinine (95%CI: 1.0, 1.3). An inverse association between urine BPA level and height was observed in boys. After adjustment for potential confounders, height *Z* score at enrolment in boys decreased by 0.49 for the highest exposure level (above 10.9 μg/g creatinine as the 90th percentile), compared with the lowest BPA exposure (below 0.2 μg/g creatinine as the 25th percentile) (95%CI: −0.96, −0.01; *p*-trend = 0.024). The inverse association remained between BPA exposure and height *Z* score at visit 2. The GEE model showed that a 1-unit increase in log_10_-transformed BPA concentrations was associated with a 0.15-point decrease in height *Z* score over the follow-up (95%CI: −0.30, −0.01). BPA was not associated with height growth in girls.

**Conclusions:**

Our findings indicate an inverse association between urine BPA level and height growth in boys. These findings need to be confirmed in further studies.

## Introduction

Bisphenol A (BPA) is a synthetic compound used extensively worldwide in the production of polycarbonate plastics and epoxy resins, which are found in many consumer products (e.g., water bottles, water piping, the lining of tin cans, toys, and thermal receipt paper) [1, 2]. Due to the ubiquitous presence of BPA in the environment and its known toxicological implications [3], especially considering children who are more vulnerable to environmental exposures, there have been increasing concerns regarding the potential impacts of BPA on children’s health.

Linear growth is one of the critical markers of the overall health status of a child [4]. It occurs in tandem with pubertal development, with the activation of hypothalamic pituitary gonadal axis as the proposed driver of the pubertal growth spurt [5]. Before puberty, growth velocity gradually decelerates slightly [6]. Then, growth velocity accelerates when puberty begins and reaches a peak on average 22 months after initiation [7]. This accelerated growth period of puberty, which is influenced by many hormones and growth factors, accounts for about 20% of final adult height [7]. Sex steroids play a crucial role in pubertal growth both at the systemic level via the growth hormone/insulin-like growth factor-I (GH/IGF-I) axis and at the local level of the epiphyseal growth plate [7]. Pituitary growth hormone secretion increases during puberty in response to sex steroids [8]. Besides, sex steroids are likely to have a direct action on chondrocytes because the androgen receptor (AR) and both oestrogen receptor α (ERα) and β (ERβ), have been demonstrated in growth plate tissue at the mRNA and protein level in several species, including human [7].

As an endocrine-disrupting chemical, BPA can act through a variety of physiological receptors, including ERs and AR. Previous studies reported that BPA could disrupt pubertal development through its oestrogenic and antiandrogenic effects [3, 9, 10]. However, there was scarce evidence with regard to the impact of BPA on longitudinal bone growth in puberty. In this study, we examined the impact of BPA exposure on pubertal growth in height among school-aged children and any sex-specific effect, since gender differences were reported in studies examining BPA and growth outcomes, such as gestational length and birth weight [11].

## Methods

This was an ancillary study of a national survey of pubertal development and adolescent health in China. The national survey was designed to collect anthropometric measures and related information in order to assess pubertal growth and development. The current study was conducted during 2011–2012 among school-aged children in Jiading district, a highly industrialised district located in the northwestern part of Shanghai, with middle level of population density and income. It was selected as one of the sites of the national survey. This study added collection of urine samples at enrolment and ascertained additional questions concerning factors related to pubertal growth when the students were first enroled (visit 1) and subsequently visited at 19 months after enrolment (visit 2). A detailed description of the study has been presented previously [12]. The following are methods relevant to the present study.

### Study population

Three large schools (one elementary, one middle, and one high school) in Jiading district participated in the study. All students aged 9–18 years in grades 4 through 12 were eligible for inclusion in the study. Four classes of students from each grade were randomly selected and recruited with approximately 160 students from each grade. Among 1451 eligible students, 18 (1.2%) refused to participate. An additional 90 students did not provide urine samples, of whom 72 were girls due to menstruation at the time of specimen collection. Samples from 17 students were accidentally damaged during transportation. Thus, 1326 students were included at visit 1, consisting of 91.4% of the initial eligible population. At visit 2, 414 students were no longer available as they had graduated from the participating school. In addition, 158 students did not complete the physical examinations during visit 2. Thus, 754 participants had information for both visits, and were included in the final analyses.

The study was approved by the committees for protection of human subjects at Shanghai Institute of Planned Parenthood Research and School of Public Health, Fudan University. Parents of all students were sent a consent form with a detailed description of the study prior to enrolment. Parents were asked to inform the teachers if they did not want their children to participate in the study. All students were also informed by their teachers of the study purpose, process and the voluntary nature of participation in advance, and reminded again at the time of data collection.

### BPA exposure assessment

Spot urine samples collected at visit 1 were measured for total urine BPA concentrations (free plus conjugated species) by high-performance liquid chromatography (HPLC) based on the modified methods of Yang et al. [13], as described in previous studies [12, 14]. In brief, the reaction mixtures of phosphorous acid buffer, β-glucuronidase (Sigma Chemical Co., St. Louis, MO) and sample aliquots in glass tubes were incubated for hydrolyzation, and then were extracted twice with ether:*n*-hexane (1:1) (HPLC grade, Dikma). The supernatants were evaporated with nitrogen gas. The residue was dissolved in 40% acetonitrile (HPLC grade, Dikma) and analysed by HPLC with fluorescence detection. Analysis was conducted at the Department of Occupational Health and Toxicology, School of Public Health & WHO Collaborating Center for Occupational Health, Fudan University, Shanghai, China. Laboratory techniques and quality control protocols have been reported previously [14]. The limit of detection (LOD) for BPA was 0.31 μg/L, which is comparable to the published LOD of 0.4 μg/L [15]. Values below LOD were replaced with LOD/√2 as the standard practice. All BPA concentrations were accounted for urine creatinine levels to control for urine dilution. Due to the potential non-monotonic dose response effects of BPA [16], we used 25th, 50th, and 75th percentile of creatinine-adjusted BPA concentrations (micrograms per gram creatinine) as cutoff points. In addition to quartiles, we used 90th percentile to obtain a categorical variable and examined the effects of relatively high BPA exposure in our population. Further, we included log_10_-transformed creatinine as an independent variable in models using log_10_-transformed BPA concentrations as a continuous variable, as recommended by Barr et al. [17].

### Height growth and pubertal assessment

For each participant, a trained physician conducted standardised physical examinations without knowledge of the child’s BPA exposure status. At visits 1 and 2, height was measured with the participants barefoot, in line with recommendations from the National Health and Nutrition Examination Survey [18]. Age- and sex-specific *Z* scores for height at each visit were calculated based on references from a previous study in a similar study population (height *Z* score = [participant’s height−sex- and age-specific population height mean]/sex- and age-specific population height standard deviation) [19]. Pubertal development status, which might represent a distinct growth and/or hormonal profile, was measured according to the internationally accepted Tanner stage criteria [20]. Pubic hair development at visit 1 was graded as 1 (pre-puberty), 2 (onset), 3 (ongoing), 4 (nearly complete) or 5 (complete and adult-like). Participants at pubic hair Tanner stage 1 were classified as prepubertal, while those at Tanner stage 2 or higher (Tanner stage 2+) were classified into the pubertal group.

### In-person information collection

At visits 1 and 2, the following information was collected by self-administered questionnaires from children and their parents: (1) demographic characteristics, including children’s sex and age, parental age and height, maternal education level (≥ college vs. < college). In addition, a 1–5 rating scale (poor to good) was used for self-evaluation of household income. Those who reported 1–3 were categorised as middle and below, while 4–5 were categorised as upper middle. (2) Other maternal characteristics, including gestational age, parity, single or multiple births, prenatal passive smoking (yes/no), and exclusive breastfeeding (yes/no). (3) Information on children’s dietary intake was collected using a food frequency questionnaire validated in a similar Chinese population [21] to ascertain the frequency of food intake per week, including junk foods, fish, meat, dairy products, fruits and vegetables, and soy-based foods. The participants were asked to self-evaluate whether they have an unbalanced diet (yes/no). (4) Time spent on physical activity were collected and categorised as ≥ 30 min/day and < 30 min/day. (5) Children’s current depression status was assessed using the published Children’s Depression Inventory [22]. (6) Children’s exposure status to environmental tobacco smoke (ETS) was collected by asking whether their father or mother was a smoker (yes/no).

### Statistical analyses

Due to the sexual dimorphic pattern of pubertal growth [7], as well as potential sex difference in the effects of BPA [11, 23], we examined height growth in relation to peripubertal BPA exposure in boys and girls separately. Initially, linear regression models were used to examine the association between urine BPA level and height *Z* score at visits 1 and 2, respectively. Beta coefficients were calculated to represent the change in height *Z* score for each unit increase of BPA variable. Then, in order to assess the association between BPA exposure and height growth over the follow-up, generalised estimating equation (GEE) models were used to account for the correlation between repeated height *Z* scores obtained for two visits from the same child [24]. We characterised creatinine-adjusted urine BPA concentrations as a categorical variable in our statistical models. In addition, we used a continuous log-transformed BPA variable in models including log-creatinine as a covariate. As a sensitivity analysis, we stratified main analyses by pubic hair development status at visit 1 (Tanner stage 1 vs. Tanner stage 2+), since puberty might represent a distinct growth and/or hormonal profile [6, 8] and thus could be a modifier for the association between BPA exposure and height growth. In addition, pubertal growth spurt in both boys and girls begins at pubic hair stage 2 [6]. We also explored the potential modifying effect of pubertal status on the association by entering the interaction term (BPA*pubertal status) in the models, although our study sample size is likely underpowered to study interactions.

We evaluated a wide range of covariates, including parental characteristics, such as household income, paternal age, paternal height (continuous variable), maternal age, maternal height (continuous variable), maternal education, gestational age, parity, single or multiple births, prenatal passive smoking, and exclusive breastfeeding, and child characteristics, such as sex, age (continuous variable), pubertal status, unbalanced diet, physical activity, depression score, dietary intake, and ETS. We first constructed a basic model adjusted for child sex and age at visit 1, and then added each covariate one at the time to the basic model. We retained in the final multivariable-adjusted models only those covariates that altered the coefficient of the BPA exposure by >10%.

We repeated all analyses excluding children with urine creatinine values < 0.2 g/L, to reduce the influence of very dilute specimens (*n* = 4) [25]. We also restricted analysis to first births, single births or term births, to examine whether the results were stable, since these characteristics could be related to different growth patterns [26–29]. All statistical analyses were performed using Stata 12.0 (Stata Corp., LP, College Station, TX). Statistical significance was defined as a *p*-value < 0.05.

## Results

Participants included in analyses (*n* = 754) had a mean (±SD) age of 12.9 (±2.3) years. At enrolment, a total of 454 children (60.3%) had reached Tanner stage 2+ for pubic hair development (Table 1), with a mean (±SD) age of 14.2 (±1.8) years, while the mean age of children at Tanner stage 1 was 10.8 (±1.3) years (*p* < 0.01). The mean height of children by age group revealed an expected chronological order, increasing reasonably from visit 1 to visit 2 (Table 2). The geometric mean of urine BPA concentrations was 1.6 μg/L (95%CI: 1.4, 1.8) or 1.2 μg/g creatinine (95%CI: 1.0, 1.3). The median of creatinine-adjusted BPA concentrations was 1.3 (0.2, 4.9) μg/g creatinine, while the 90th percentile was 10.9 μg/g creatinine. Lower urine BPA concentrations were found in children with higher paternal age (Table S1). BPA concentrations did not vary according to other characteristics in Table 1.

**Table 1:** Characteristics of participants (*n*=754)

Characteristics	*N* (%)	Mean (SD)
*Child characteristics*		
Age at visit 1		12.9 (2.3)
Male sex (%)	370 (49.1)	
Tanner stage 2 or higher at visit 1 (%)	454 (60.3)	
Parity ≥2 (%)	39 (5.3)	
Gestational age (weeks)		39.6 (1.4)
Preterm (%)	29 (3.9)	
Singleton (%)	734 (98.5)	
Breastfeeding exclusive (%)	375 (51.2)	
*Life style and mental health*		
Unbalanced diet (%)	307 (44.2)	
Physical activity ≥30 min/day (%)	304 (41.1)	
Depression score^a^ ≥10 (%)	357 (57.5)	
*Dietary intake*		
Junk foods intake ≥5 days/week (%)	186 (24.8)	
Fish intake ≥5 days/week (%)	185 (24.6)	
Meat intake ≥5 days/week (%)	66 (8.8)	
Dairy products intake everyday (%)	331 (44.0)	
Fruits and vegetables intake everyday (%)	557 (74.3)	
Soy-based foods intake everyday (%)	184 (24.4)	
*Parental characteristics*		
Maternal age		24.5 (3.3)
Maternal height		160.2 (4.5)
Paternal age		26.8 (3.7)
Paternal height		172.3 (4.7)
Parental tobacco smoke positive (%)	496 (66.7)	
Prenatal passive smoking (%)	126 (17.1)	
Maternal education ≥ college (%)	308 (41.5)	
Household income > middle (%)	247 (33.0)	

^a^The *CDI* (Children’s Depression Inventory) was used to assess depression status. Higher scores represent higher depressive symptoms

**Table 2:** Mean height by age of children (with SD) at two visits

Age group (at visit 1)	Girls’ height (cm)			Boys’ height (cm)		
	*N*	Visit 1	Visit 2	*N*	Visit 1	Visit 2
9–10	83	140.9 (6.7)	151.8 (6.9)	83	139.2 (7.0)	148.2 (8.0)
11	51	151.4 (6.7)	158.9 (5.3)	61	150.4 (8.5)	163.6 (7.9)
12	73	155.9 (5.4)	160.0 (5.2)	80	158.7 (9.1)	168.9 (6.7)
13–14	68	158.7 (7.2)	161.1 (6.7)	72	166.7 (7.3)	173.3 (5.9)
15	43	162.8 (4.8)	163.3 (5.1)	33	172.9 (5.6)	175.1 (5.2)
16–17	66	163.0 (4.8)	163.2 (4.8)	41	174.8 (6.2)	175.4 (6.4)

There was an inverse association between urine BPA level and boy’s height *Z* score (Table 3). In analyses according to continuous BPA exposure variable, after adjustment for log-creatine and other potential confounders, the β coefficients for the association between BPA (log_10_-transformed, in μg/L) and boy’s height *Z* score were −0.18 (95%CI: −0.34, −0.01) for visit 1 and −0.13 (95%CI: −0.28, 0.02) for visit 2 (Table 3). The results of models with percentile of urine BPA concentrations were consistent with those using continuous variable. At visit 1, compared with the lowest BPA exposure, height *Z* score in boys decreased by 0.49 at the exposure level above 90th percentile (10.9 μg/g creatinine) (adjusted *β* = −0.49; 95%CI: −0.96, −0.01; *p*-trend = 0.024). An inverse but a bit weaker association remained at visit 2 (adjusted *β* = −0.38 for the highest level vs. the lowest one; 95%CI: −0.83, 0.06). However, urine BPA was not associated with height *Z* score in girls.

**Table 3:** Associations between urine BPA level and height *Z* score at two visits^a^

	Percentiles of urine BPA concentrations (μg/g Cr)					*p*-trend	Log_10_-BPA (μg/L)
	<25th	25–50th	50–75th	75–90th	≥90th		
*Boys* (*n*=*370*)							
Visit 1							
Crude	Ref	−0.02 (−0.34,0.30)	−0.12 (−0.44,0.20)	0.01 (−0.35,0.38)	−0.53** (−**0.93**,−**0.12**)	0.058*	−0.11 (−0.25,0.03)
Adjusted^b^	Ref	−0.04 (−0.38,0.30)	−0.33* (−**0.67**,**0.01**)	−0.20 (−0.60,0.21)	−0.49** (−**0.96**,−**0.01**)	0.024**	−0.18** (−**0.34**,−**0.01**)
Visit 2							
Crude	Ref	0.09 (−0.20,0.37)	−0.05 (−0.33,0.24)	0.09 (−0.24,0.42)	−0.39** (−**0.76**,−**0.03**)	0.143	−0.08 (−0.21,0.05)
Adjusted^b^	Ref	0.07 (−0.25,0.38)	−0.26 (−0.58,0.06)	−0.06 (−0.44,0.33)	−0.38* (−**0.83**,**0.06**)	0.069*	−0.13* (−**0.28**,**0.02**)
*Girls* (*n*=*384*)							
Visit 1							
Crude	Ref	−0.06 (−0.35,0.24)	−0.14 (−0.43,0.16)	0.08 (−0.26,0.42)	−0.07 (−0.47,0.33)	0.964	−0.01 (−0.15,0.13)
Adjusted^b^	Ref	−0.05 (−0.33,0.22)	−0.05 (−0.33,0.22)	0.07 (−0.24,0.39)	0.08 (−0.31,0.46)	0.552	0.04 (−0.09,0.18)
Visit 2							
Crude	Ref	−0.003 (−0.30,0.29)	−0.12 (−0.41,0.18)	−0.07 (−0.41,0.26)	0.01 (−0.39,0.41)	0.692	−0.05 (−0.19,0.09)
Adjusted^b^	Ref	0.02 (−0.25,0.29)	−0.05 (−0.32,0.22)	−0.07 (−0.39,0.24)	0.13 (−0.24,0.51)	0.962	−0.02 (−0.16,0.11)

^a^Beta coefficients were calculated to represent the change in height *Z* score for each unit of increase of BPA variable

^b^Models with categorised BPA variable adjusted for: age, maternal education, paternal age, maternal height, paternal height, singleton, pubertal status, unbalanced diet, sports activity, depression, and junk foods; models with continuous BPA variable further adjusted for log-creatinine

*p<0.1, **p<0.05

The GEE multivariable model (Table 4) assesses the mean height over the two visits in relation to urine BPA and shows the similar results. In boys, peripubertal exposure to BPA showed a significant inverse association with height *Z* score, which decreased by 0.15 per log_10_-transformed μg/L BPA increase (95%CI: −0.30, −0.01). We explored the time effect on the association between BPA and height growth by adding to the GEE model an interaction term (BPA*visit). However, no significant interaction was found for the product term BPA*visit (*p*-value = 0.455) in the model for boys (data not shown).

**Table 4:** GEE regression model results for associations between urine BPA level and height *Z* score over the follow-up^a^

	Percentiles of urine BPA concentrations (μg/g Cr)					*p*-trend	Log_10_-BPA (μg/L)	
	25–50th	25–50th	50–75th	75–90th	≥90th			
Boys (*n*=370)								
Crude	Ref	0.03 (−0.25,0.32)	−0.08 (−0.37,0.20)	0.05 (−0.28,0.38)	−0.46** (−**0.82**,−**0.09)**	**0.074**	−0.09 (−0.22,0.04)	
Adjusted^b^	Ref	0.01 (−0.29,0.31)	−0.29* (−**0.60**,**0.01)**	−0.13 (−0.49,0.24)	−0.44** (−**0.85**,−**0.02)**	**0.026**	−0.15** (−**0.30**,−**0.01)**	
Girls (*n*=384)								
Crude	Ref	−0.03 (−0.31,0.25)	−0.13 (−0.41,0.15)	0.004 (−0.32,0.33)	−0.03 (−0.42,0.35)	0.820	−0.03 (−0.17,0.11)	
Adjusted^b^	Ref	−0.01 (−0.27,0.24)	−0.05 (−0.30,0.20)	0.0001 (−0.29,0.29)	0.11 (−0.25,0.46)	0.730	0.01 (−0.12,0.13)	

^a^Beta coefficients were calculated to represent the change in height *Z* score for each unit of increase of BPA variable

^b^Models with categorised BPA variable adjusted for: age, maternal education, paternal age, maternal height, paternal height, singleton, pubertal status, unbalanced diet, sports activity, depression, and junk foods; models with continuous BPA variable further adjusted for log-creatinine

*p<0.1, **p<0.05

Given that 60.3% of participants were at Tanner stage 2+, which might have a distinct growth and/or hormonal profile, Table 5 presents the association between urine BPA and height *Z* score in boys stratified by pubertal status. At visit 1, while no association was found among boys at Tanner stage 1, per log_10_-transformed BPA increase was associated with a significantly lower height *Z* score (adjusted *β* = −0.27; 95%CI: −0.48, −0.05) among those at Tanner stage 2+. However, no significant interaction was found when we added the term BPA*pubertal status to the model (*p*-value = 0.138). Urine BPA was not associated with girl's height regardless of whether she had reached Tanner stage 2 or not (Table S2).

**Table 5:** Associations between urine BPA level and height *Z* score in boys stratified by pubertal status^a^

	Percentiles of urine BPA concentrations (μg/g Cr)					*p*-trend	Log_10_-BPA (μg/L)
	<25th	25–50th	50–75th	75–90th	≥90th		
*Tanner stage 1* (*n=187*)							
Visit 1							
Crude	Ref	−0.02 (−0.45,0.41)	−0.13 (−0.58,0.32)	0.32 (−0.19,0.84)	−0.27 (−0.80,0.26)	0.787	−0.04 (−0.23,0.16)
Adjusted^b^	Ref	0.01 (−0.53,0.55)	−0.17 (−0.75,0.41)	0.30 (−0.38,0.99)	−0.16 (−0.90,0.59)	0.985	−0.02 (−0.28,0.24)
Visit 2							
Crude	Ref	0.25 (−0.17,0.66)	−0.003 (−0.43,0.43)	0.34 (−0.15,0.83)	−0.25 (−0.76,0.25)	0.620	−0.06 (−0.25,0.12)
Adjusted^b^	Ref	0.28 (−0.22,0.79)	−0.15 (−0.69,0.39)	0.23 (−0.42,0.87)	0.05 (−0.65,0.74)	0.970	−0.03 (−0.28,0.21)
*Tanner stage 2 or higher* (*n=183*)							
Visit 1							
Crude	Ref	0.02 (−0.43,0.46)	−0.14 (−0.58,0.30)	−0.30 (−0.80,0.20)	−0.74** (−**1.36**,−**0.13)**	**0.017**	−0.22** (−**0.44**,−**0.01)**
Adjusted^b^	Ref	−0.01 (−0.44,0.42)	−0.43** (−**0.86**,−**0.01)**	−0.45* (−**0.94**,**0.04)**	−0.35 (−1.00,0.29)	**0.025**	−0.27** (−**0.48**,−**0.05)**
Visit 2							
Crude	Ref	−0.08 (−0.47,0.32)	−0.09 (−0.48,0.30)	−0.14 (−0.59,0.30)	−0.54* (−**1.09**,**0.003)**	0.102	−0.10 (−0.29,0.09)
Adjusted^b^	Ref	0.003 (−0.38,0.39)	−0.25 (−0.62,0.13)	−0.14 (−0.58,0.30)	−0.22 (−0.79,0.36)	0.229	−0.11 (−0.30,0.08)

^a^Beta coefficients were calculated to represent the change in height *Z* score for each unit of increase of BPA variable

^b^Models with categorised BPA variable adjusted for: age, maternal education, paternal age, maternal height, paternal height, singleton, unbalanced diet, sports activity, depression, and junk foods; models with continuous BPA variable further adjusted for log-creatinine

*p<0.1, **p<0.05

In sensitivity analyses, excluding children with urine creatinine values < 0.2 g/L did not influence results (data not shown). After restricting the analysis to first births, single births or term births, results did not change meaningfully (data not shown).

## Discussion

This longitudinal follow-up study examined the relationship between exposure to BPA and growth of height in school aged children. We observed that urine BPA was inversely associated with height *Z* score in boys. To our knowledge, this is the first study that appeared to show growth suppression by BPA exposure among peripubertal boys. Although the impact of BPA on longitudinal bone growth in puberty has not been well examined, impact of BPA on bone development in early life has been reported in animal studies. In-utero exposure to high doses of BPA (1000 mg/kg/day from days 1 to 20 of gestation) adversely impacted the bone turnover and skeletal development in rats [30], while developmental BPA exposure at low doses (10 μg/kg/day from gestational day 11 to postnatal day 12) increased femur length in male mice [31]. Although environmentally relevant BPA exposure is usually much lower than toxicologically level in animal studies [32], previous studies have reported adverse effects of environmental BPA exposure on human health outcomes [33], including pubertal development [9, 10]. In addition, an epidemiological study assessing foetal growth has shown that prenatal exposure to environmental BPA was associated with reduced femur length from 12 to 20 weeks [34].

Although the biological explanation behind the association between BPA exposure and growth in height is unclear at this point, BPA has been shown to affect a variety of hormone functions and can potentially disrupt height growth. For example, BPA can interact with oestrogen receptors to act as an oestrogen agonist or antagonist. BPA can also directly bind to androgen receptors and is possibly antiandrogenic, blocking endogenous androgen action. Animal studies also showed that BPA can induce GH transcription and release [35], and alter IGF-I concentration [36]. Both sex steroids and GH/IGF-I axis play crucial roles in regulating longitudinal growth and skeletal maturation [7, 8].

Significant associations between urine BPA and depressed height growth were found in boys, but not in girls. Our results are consistent with previous studies which observed sex-specific associations of prenatal BPA with anthropometric measures. These studies reported that maternal urinary BPA concentrations were significantly associated with foetal growth in males, but not females [23, 34]. Many other studies also reported sex differences in effects of BPA [12, 37, 38]. In both human and animal populations, responsiveness to oestrogenic endocrine-disrupting chemicals is known to be variable across individuals, and it is possible that endogenous sex hormones play a role [39], which also may explain sex-specific effects of BPA.

Although the inverse association between BPA and height *Z* score in boys at visit 1 remained after the follow-up period, it was weaker and non-significant at visit 2. This finding, combined with our earlier publication indicating BPA associated with accelerated pubertal onset in boys [9], is consistent with previous findings on the relationship between pubertal timing and height growth, which reported that children who matured earlier tended to be shorter at a given age than those who matured later, although, on average, final heights at full maturity did not differ [40, 41]. However, no significant interaction was found when we examined the time effect on the association between BPA and pubertal height growth, likely due to the limited statistical power from our sample size. Further investigation in future studies are needed to draw a conclusion about the potential catch-up growth phenomenon.

In the present study, results also suggest potential differences in the BPA-height growth associations according to pubertal status. In stratified analyses (Table 5), a strengthened association towards higher urine BPA and lower height *Z* score was found in pubertal boys, while no significant association was observed in prepubertal boys. A potential explanation is that puberty with rapid linear growth and sex maturation, is a hormone-dependent stage of development and is more vulnerable to endocrine disruption [42]. Although our sample size might be underpowered to study interactions, we explored the potential modifying effect of pubertal status, and the product term BPA*pubertal status did not reach the statistical significance (*p*-value = 0.138). Nevertheless, these findings should be cautiously treated because we were not able to follow the exact trajectories of height growth according to pubertal status in the current study.

There are a few potential limitations of our study. One consideration is about BPA exposure. The mean concentration of urine BPA measured in this study is comparable to those reported previously in similar Chinese populations [43, 44]. However, a single spot urine sample collected in our study may represent only recent exposure due to the relatively short half-life of BPA, and subsequently could have led to non-differential misclassification of BPA exposure, resulting in attenuation of the observed association. An additional consideration is that the peripubertal period is likely not the only critical window of exposure to factors which alter pubertal growth. Early-life exposure can also contribute to late development [16]. Therefore, a lack of information on BPA exposure during other critical windows makes it difficult to rule out the impact of BPA exposure at earlier life stages on pubertal height growth. Finally, this study was limited by its insufficient length of follow-up and height measures to follow the exact trajectorie of height growth, which made further analyses less conclusive.

A main strength of our study is its longitudinal follow-up study design. With children’s height measures at two visits, this study provided an ability to examine not only the association between BPA exposure and reduced pubertal height, but also the association potentially modified by time and pubertal status. However, the insufficient length of follow-up and height measures of this study limited its ability to examine how BPA exposure would alter height growth trajectory in a peripubertal period. Thus, long-term longitudinal studies are needed to clarify our findings. Another strength is that the present study collected information on a wide range of children’s and parental characteristics as potential confounders, especially dietary patterns and food intake. Our estimates did not change noticeably after inclusion of a variety of potential confounders, suggesting robustness for the observed associations.

Another consideration is about urine creatinine concentrations which we used to control for urine dilution. Both creatinine and specific gravity are common methods for adjusting dilution. A study which compared creatinine with specific gravity suggested the correction method of specific gravity is preferred to evaluate BPA exposure for children [44], since specific gravity might be less affected by physiological parameters, such as age, sex, BMI, muscle mass, etc. [17, 44, 45]. However, a study reported that, like creatinine, specific gravity differed across age groups due to variations in muscle mass [46]. Therefore, multivariate control for dilution factors as independent variables may be a better method to compare biomarker concentrations between individuals than when modelling the marker as a ratio [17, 47]. In this study, we also included creatinine as a variable in the model as recommended by Barr et al. [17], which would allow our results to be compared with other studies adjusting for specific gravity in the future.

## Conclusions

Our findings provide first epidemiological evidence that higher environmental BPA exposure is associated with reduced height growth in boys. Given this new finding, further studies, especially long-term longitudinal studies, are needed to verify these results.

## Electronic supplementary material

SupplementaryTables

## References

[1] Hoekstra EJ, Simoneau C (2013). Release of bisphenol A from polycarbonate: a review. Crit Rev Food Sci Nutr.

[2] Vandenberg LN, Hauser R, Marcus M, Olea N, Welshons WV (2007). Human exposure to bisphenol A (BPA). Reprod Toxicol.

[3] Peretz J, Vrooman L, Ricke WA, Hunt PA, Ehrlich S, Hauser R (2014). Bisphenol A and reproductive health: update of experimental and human evidence, 2007–2013. Environ Health Persp.

[4] Andrea G, Achamyeleh G, Lee JM (2015). Relationship between timing of peak height velocity and pubertal staging in boys and girls. J Clin Res Pediatr Endocrinol.

[5] Ebling FJP (2005). The neuroendocrine timing of puberty. Reproduction.

[6] Bordini B, Rosenfield RL (2011). Normal pubertal development: part II: clinical aspects of puberty. Pediatr Rev / Am Acad Pediatr.

[7] Perry RJ, Farquharson C, Ahmed SF (2008). The role of sex steroids in controlling pubertal growth. Clin Endocrinol (Oxf).

[8] Bordini B, Rosenfield RL (2011). Normal pubertal development: Part I: The endocrine basis of puberty. Pediatr Rev / Am Acad Pediatr.

[9] Wang Z, Li D, Miao M, Liang H, Chen J, Zhou Z (2017). Urine bisphenol A and pubertal development in boys. Int J Hyg Environ Health.

[10] Miao M, Wang Z, Liu X, Liang H, Zhou Z, Tan H (2017). Urinary bisphenol A and pubertal development in Chinese school-aged girls: a cross-sectional study. Environ Health: A Glob Access Sci Source.

[11] Veiga-Lopez A, Kannan K, Liao C, Ye W, Domino SE, Padmanabhan V (2015). Gender-specific effects on gestational length and birth weight by early pregnancy BPA exposure. J Clin Endocrinol Metab.

[12] Li DK, Miao M, Zhou Z, Wu C, Shi H, Liu X (2013). Urine bisphenol-A level in relation to obesity and overweight in school-age children. PLoS One.

[13] Yang M, Kim SY, Lee SM, Chang SS, Kawamoto T, Jang JY (2003). Biological monitoring of bisphenol a in a Korean population. Arch Environ Contam Toxicol.

[14] He Y, Miao M, Herrinton LJ, Wu C, Yuan W, Zhou Z (2009). Bisphenol A levels in blood and urine in a Chinese population and the personal factors affecting the levels. Environ Res.

[15] Calafat AM, Ye X, Wong LY, Reidy JA, Needham LL (2008). Exposure of the U.S. population to bisphenol A and 4-tertiary-octylphenol: 2003–2004. Environ Health Perspect.

[16] Vandenberg LN, Maffini MV, Sonnenschein C, Rubin BS, Soto AM (2009). Bisphenol-A and the great divide: a review of controversies in the field of endocrine disruption. Endocr Rev.

[17] Barr DB, Wilder LC, Caudill SP, Gonzalez AJ, Needham LL, Pirkle JL (2005). Urinary creatinine concentrations in the U.S. population: implications for urinary biologic monitoring measurements. Environ Health Perspect.

[18] NHANES. National Health and Nutrition Examination Survey. Anthropometry Procedures Manual (2007). http://www.cdc.gov/nchs/data/nhanes/nhanes_07_08/manual_an.pdf. Accessed 18 Sep 2013.

[19] Jiang YF, Cole T, Pan HQ, Lin ZF, Ju MF, Zhang L (2007). Construction of height and weight percentile references for Shanghai children and adolescents. Shanghai J Prev Med.

[20] Tanner JM, Whitehouse RH (1976). Clinical longitudinal standards for height, weight, height velocity, weight velocity, and stages of puberty. Arch Dis Child.

[21] Hsu-Hage BH, Wahlqvist ML (1992). A food frequency questionnaire for use in Chinese populations and its validation. Asia Pac J Clin Nutr.

[22] Kovacs M (1992). Children depression inventory CDI: Manual.

[23] Lee BE, Park H, Hong YC, Ha M, Kim Y, Chang N (2014). Prenatal bisphenol A and birth outcomes: MOCEH (Mothers and Children’s Environmental Health) study. Int J Hyg & Environ Health.

[24] Hardin JW, Hilbe JM. Generalized linear models and extensions. 3rd ed. College Station: Stata Press; 2012.

[25] Wolff MS, Teitelbaum SL, Pinney SM, Windham G, Liao L, Biro F (2010). Investigation of relationships between urinary biomarkers of phytoestrogens, phthalates, and phenols and pubertal stages in girls. Environ Health Persp.

[26] Ong KK, Preece MA, Emmett PM, Ahmed ML, Dunger DB (2002). Size at birth and early childhood growth in relation to maternal smoking, parity and infant breast-feeding: longitudinal birth cohort study and analysis. Pediatr Res.

[27] Grantz KL, Grewal J, Albert PS, Wapner R, D’Alton ME, Sciscione A (2016). Dichorionic twin trajectories: the NICHD Fetal Growth Studies. Am J Obstet Gynecol.

[28] Valvi D, Casas M, Mendez MA, Ballesteros-Gomez A, Luque N, Rubio S (2013). Prenatal bisphenol a urine concentrations and early rapid growth and overweight risk in the offspring. Epidemiology.

[29] Jedrychowski WA, Perera FP, Majewska R, Mrozek-Budzyn D, Mroz E, Roen EL (2015). Depressed height gain of children associated with intrauterine exposure to polycyclic aromatic hydrocarbons (PAH) and heavy metals: the cohort prospective study. Environ Res.

[30] Kim JC, Shin HC, Cha SW, Koh WS, Chung MK, Han SS (2001). Evaluation of developmental toxicity in rats exposed to the environmental estrogen bisphenol A during pregnancy. Life Sci.

[31] Pelch KE, Carleton SM, Phillips CL, Nagel SC (2011). Developmental exposure to xenoestrogens at low doses alters femur length and tensile strength in adult mice. Biol Reprod.

[32] EFSA CEF Panel (EFSA Panel on Food Contact Materials E, Flavourings and Processing Aids). Scientific opinion on the risks to public health related to the presence of bisphenol A (BPA) in foodstuffs: executive summary. EFSA J. 2015;13:3978, 23pp.

[33] Rochester JR (2013). Bisphenol A and human health: a review of the literature. Reprod Toxicol.

[34] Casas M, Valvi D, Ballesterosgomez A, Gascon M, Fernández MF, Garciaesteban R (2016). Exposure to bisphenol A and phthalates during pregnancy and ultrasound measures of fetal growth in the INMA-sabadell cohort. Environ Health Persp.

[35] Dang VH, Nguyen TH, Lee GS, Choi KC, Jeung EB (2009). In vitro exposure to xenoestrogens induces growth hormone transcription and release via estrogen receptor-dependent pathways in rat pituitary GH3 cells. Steroids.

[36] Ramirez MC, Bourguignon NS, Bonaventura MM, Lux-Lantos V, Libertun C, Becu-Villalobos D (2012). Neonatal xenoestrogen exposure alters growth hormone-dependent liver proteins and genes in adult female rats. Toxicol Lett.

[37] Braun JM, Kalkbrenner AE, Calafat AM, Yolton K, Ye X, Dietrich KN (2011). Impact of early-life bisphenol A exposure on behavior and executive function in children. Pediatrics.

[38] Xu X, Tian D, Hong X, Chen L, Xie L (2011). Sex-specific influence of exposure to bisphenol-A between adolescence and young adulthood on mouse behaviors. Neuropharmacology.

[39] Howdeshell KL, Hotchkiss AK, Thayer KA, Vandenbergh JG, vom Saal FS (1999). Exposure to bisphenol A advances puberty. Nature.

[40] Vizmanos B, Martí‐Henneberg C, Clivillé R, Moreno A, Fernández‐Ballart J (2001). Age of pubertal onset affects the intensity and duration of pubertal growth peak but not final height. Am J Hum Biol.

[41] Llop-Vinolas D, Vizmanos B, Closa Monasterolo R, Escribano Subias J, Fernandez-Ballart JD, Marti-Henneberg C (2004). Onset of puberty at eight years of age in girls determines a specific tempo of puberty but does not affect adult height. Acta Paediatr.

[42] Fudvoye J, Bourguignon JP, Parent AS (2014). Endocrine-disrupting chemicals and human growth and maturation: a focus on early critical windows of exposure. Vitam Horm.

[43] Zhang M, Duan Z, Wu Y, Liu Z, Li K, Wang L (2015). Occurrence and profiles of the artificial endocrine disruptor bisphenol A and natural endocrine disruptor phytoestrogens in urine from children in China. Int J Environ Res & Public Health.

[44] Wang B, Wang H, Zhou W, He Y, Zhou Y, Chen Y (2014). Exposure to bisphenol A among school children in eastern China: a multicenter cross-sectional study. J Expo Sci & Environ Epidemiol.

[45] Sauvé JF, Lévesque M, Huard M, Drolet D, Lavoué J, Tardif R (2015). Creatinine and specific gravity normalization in biological monitoring of occupational exposures. J Occup & Environ Hyg.

[46] Moriguchi J, Ezaki T, Tsukahara T, Fukui Y, Ukai H, Okamoto S (2005). Decreases in urine specific gravity and urinary creatinine in elderly women.. International Archives of Occupational & Environ Health.

[47] Muscat JE, Liu A, Richie JP (2011). A comparison of creatinine vs. specific gravity to correct for urinary dilution of cotinine. Biomark: Biochem Indic Expo, Response, susceptibility Chem.

