# Supplementary material for: Bisphenol A and pubertal height growth in school-aged children
Source: J Expo Sci Environ Epidemiol. 2018 Sep 5;29(1):109–17. doi: 10.1038/s41370-018-0063-8 (PMC6760752; doi:10.1038/s41370-018-0063-8)
Supplement: Supplementary file 1 — SupplementaryTables [file 41370_2018_63_MOESM1_ESM.docx]

**Table S1**

**Urine BPA concentration**^a^ **according to participant characteristics (n=754)**

| **Covariates** | **N** | **BPA GM (95% CI)** | **BPA Median (25^th^, 75^th^ )** |
| --- | --- | --- | --- |
| **Child characteristics** |  |  |  |
| Age at visit 1 |  |  |  |
| <12 | 278 | 1.0 (0.8, 1.3) | 0.9 (0.2, 4.5) |
| 12-14 | 293 | 1.3 (1.0, 1.5) | 1.4 (0.3, 4.5) |
| ≥15 | 183 | 1.3 (1.0, 1.7) | 1.4 (0.2, 6.7) |
| Sex |  |  |  |
| Males | 370 | 1.1 (1.0, 1.4) | 1.3 (0.2, 5.0) |
| Females | 384 | 1.2 (1.0, 1.4) | 1.3 (0.2, 4.9) |
| Reach pubic hair stage 2 at visit 1 |  |  |  |
| No | 299 | 1.1 (0.9, 1.3) | 1.1 (0.2, 4.6) |
| Yes | 454 | 1.2 (1.1, 1.5) | 1.4 (0.2, 5.3) |
| Parity ≥2 |  |  |  |
| Nulliparous | 698 | 1.2 (1.0, 1.3) | 1.3 (0.2, 4.8) |
| Multiparous | 39 | 1.3 (0.7,2.4) | 1.1 (0.2, 7.0) |
| Preterm |  |  |  |
| No | 710 | 1.2 (1.0, 1.3) | 1.3 (0.2, 4.9) |
| Yes | 29 | 1.2 (0.6, 2.5) | 2.4 (0.2, 5.4) |
| Singleton |  |  |  |
| No | 11 | 2.2 (0.7, 7.0) | 3.0 (0.5, 12.2) |
| Yes | 734 | 1.2 (1.0, 1.3) | 1.3 (0.2, 4.9) |
| Breastfeeding exclusive |  |  |  |
| No | 358 | 1.1 (0.9, 1.4) | 1.2 (0.2, 4.9) |
| Yes | 375 | 1.2 (1.0, 1.5) | 1.4 (0.2, 4.9) |
| **Life style and mental health** |  |  |  |
| Unbalanced diet |  |  |  |
| No | 387 | 1.1 (0.9, 1.3) | 1.2 (0.2, 4.7) |
| Yes | 307 | 1.2 (1.0, 1.5) | 1.3 (0.2, 5.0) |
| Sports activity ≥30min/day |  |  |  |
| No | 435 | 1.2 (1.0, 1.4) | 1.3 (0.2, 4.9) |
| Yes | 304 | 1.1 (0.9, 1.4) | 1.2 (0.2, 5.0) |
| Depression score^b^ |  |  |  |
| <10 | 264 | 1.2 (1.0, 1.4) | 1.4 (0.2, 4.7) |
| ≥10 | 357 | 1.2 (1.0, 1.4) | 1.3 (0.2, 5.1) |
| **Food and nutrient intake** |  |  |  |
| Junk foods |  |  |  |
| Not regularly (<5 days/week) | 563 | 1.2 (1.0, 1.4) | 1.3 (0.2, 5.1) |
| Regularly | 186 | 1.2 (0.9, 1.6) | 1.3 (0.2, 4.9) |
| Fish |  |  |  |
| Not regularly (<5 days/week) | 567 | 1.2 (1.0, 1.3) | 1.3 (0.2, 4.7) |
| Regularly | 185 | 1.2 (0.9, 1.6) | 1.3 (0.2, 6.1) |
| Meat |  |  |  |
| Not regularly (<5 days/week) | 688 | 1.2 (1.0, 1.4) | 1.3 (0.2, 5.0) |
| Regularly | 66 | 1.1 (0.7, 1.7) | 0.7 (0.2, 4.1) |
| Dairy products |  |  |  |
| Not everyday | 421 | 1.2 (1.0, 1.4) | 1.3 (0.2, 4.8) |
| Everyday | 331 | 1.1 (0.9, 1.4) | 1.3 (0.2, 5.6) |
| Fruits and vegetables |  |  |  |
| Not everyday | 193 | 1.0 (0.8, 1.3) | 1.0 (0.2, 3.9) |
| Everyday | 557 | 1.2 (1.0, 1.4) | 1.4 (0.2, 5.3) |
| Soy-based foods |  |  |  |
| Not everyday | 569 | 1.1 (1.0, 1.3) | 1.2 (0.2, 4.5) |
| Everyday | 184 | 1.4 (1.1, 1.8) | 2.1 (0.2, 6.3) |
| **Parental Characteristics** |  |  |  |
| Maternal age |  |  |  |
| <25 | 453 | 1.1 (1.0, 1.3) | 1.3 (0.2, 4.8) |
| 25-29 | 248 | 1.2 (1.0, 1.5) | 1.3 (0.2, 5.2) |
| ≥30 | 41 | 1.1 (0.6, 2.0) | 1.3 (0.2, 6.1) |
| Maternal height |  |  |  |
| <160 cm | 290 | 1.2 (1.0, 1.5) | 1.4 (0.2, 4.9) |
| ≥160 cm | 434 | 1.1 (1.0, 1.3) | 1.2 (0.2, 4.8) |
| Paternal age |  |  |  |
| <30 | 639 | 1.2 (1.1, 1.4) |  |
| ≥30 | 102 | 0.8 (0.6, 1.2) |  |
| Paternal height |  |  |  |
| <170 cm | 156 | 1.1 (0.8, 1.4) | 1.3 (0.2, 4.3) |
| ≥170 cm | 573 | 1.2 (1.0, 1.4) | 1.3 (0.2, 5.1) |
| Environmental tobacco smoke |  |  |  |
| No | 248 | 1.1 (0.9, 1.4) | 1.2 (0.2, 4.8) |
| Yes | 496 | 1.2 (1.0, 1.4) | 1.4 (0.2, 4.9) |
| Prenatal passive smoking |  |  |  |
| No | 611 | 1.2 (1.0, 1.4) | 1.3 (0.2, 4.9) |
| Yes | 126 | 1.1 (0.8, 1.5) | 1.3 (0.2, 4.9) |
| Maternal education ≥ college |  |  |  |
| No | 435 | 1.2 (1.0, 1.4) | 1.3 (0.2, 4.9) |
| Yes | 308 | 1.1 (0.9, 1.4) | 1.3 (0.2, 4.9) |
| Household income > middle |  |  |  |
| No | 502 | 1.2 (1.0, 1.4) | 1.3 (0.2, 5.4) |
| Yes | 247 | 1.2 (0.9, 1.4) | 1.3 (0.2, 4.7) |

^a^ Creatinine-adjusted BPA (μg/g creatinine).

^b^The CDI (Children’s Depression Inventory) was used to assess depression status. Higher scores represent higher depressive symptoms.

**Table S2**

**Associations between urine BPA level and height Z score in girls stratified by pubertal status^a^**

|  | **Percentiles of urine BPA concentrations (μg/g Cr)** | | | | | **p-trend** | **Log_10_-BPA (μg/L)** |
| --- | --- | --- | --- | --- | --- | --- | --- |
|  | **<25th** | **25-50th** | **50-75th** | **75-90th** | **≥90th** |  |  |
| **Tanner stage 1 (n=112)** | | | | | | |  |
| **Visit 1** | | | | | | |  |
| Crude | Ref | -0.24 (-0.77,0.29) | -0.26 (-0.79,0.27) | -0.22 (-0.90,0.47) | 0.23 (-0.64,1.09) | 0.866 | -0.04 (-0.32,0.24) |
| Adjusted^b^ | Ref | -0.13 (-0.70,0.43) | -0.19 (-0.74,0.36) | 0.03 (-0.62,0.67) | 0.23 (-0.72,1.17) | 0.809 | 0.06 (-0.25,0.37) |
| **Visit 2** | | | | | | |  |
| Crude | Ref | -0.10 (-0.57,0.37) | -0.13 (-0.60,0.34) | -0.37 (-0.98,0.24) | 0.36 (-0.41,1.14) | 0.854 | -0.03 (-0.28,0.23) |
| Adjusted^b^ | Ref | 0.08 (-0.37,0.54) | -0.22 (-0.66,0.23) | -0.03 (-0.55,0.49) | **0.66* (-0.10,1.42)** | 0.653 | 0.01  (-0.25,0.27) |
| **Tanner stage 2 or higher (n=271)** | | | | | | |  |
| **Visit 1** | | | | | | |  |
| Crude | Ref | 0.05 (-0.31,0.41) | -0.05 (-0.41,0.31) | 0.24 (-0.17,0.64) | -0.07 (-0.53,0.40) | 0.780 | 0.02 (-0.15,0.18) |
| Adjusted^b^ | Ref | -0.02 (-0.34,0.31) | 0.03 (-0.30,0.35) | 0.10 (-0.28,0.48) | 0.02 (-0.43,0.46) | 0.664 | 0.05 (-0.11,0.21) |
| **Visit 2** | | | | | | |  |
| Crude | Ref | 0.09 (-0.27,0.45) | -0.05 (-0.41,0.30) | 0.12 (-0.28,0.53) | 0.04 (-0.42,0.51) | 0.842 | -0.01 (-0.17,0.16) |
| Adjusted^b^ | Ref | 0.01 (-0.33,0.34) | 0.03 (-0.30,0.36) | -0.06 (-0.44,0.32) | 0.03 (-0.42,0.48) | 0.957 | -0.01 (-0.17,0.15) |

a. Beta coefficients were calculated to represent the change in height Z score for each unit of increase of BPA variable.

b. Models with categorised BPA variable adjusted for: age, maternal education, paternal age, maternal height, paternal height, singleton, unbalanced diet, sports activity, depression, and junk foods; models with continuous BPA variable further adjusted for log-creatinine.

* p<0.1
